# The Effectiveness of Hyperbaric Oxygen Therapy as Salvage Treatment for Sudden Sensorineural Hearing Loss: A Retrospective Study

**DOI:** 10.7759/cureus.10819

**Published:** 2020-10-06

**Authors:** Nasser Almutairi, Ebraheem Alnofal, Amani Algouhi, Afnan S Bamajboor, Nabeel Alzaher

**Affiliations:** 1 Otolaryngology - Head and Neck Surgery, King Faisal Specialist Hospital & Research Centre, Riyadh, SAU; 2 Otolaryngology - Head and Neck Surgery, Alfaisal University College of Medicine, Riyadh, SAU; 3 Medicine, Alfaisal University College of Medicine, Riyadh, SAU; 4 Otolaryngology, King Faisal Specialist Hospital & Research Centre, Riyadh, SAU

**Keywords:** ssnhl, hbo therapy, sudden hearing loss, steroids

## Abstract

Background

Sudden sensorineural hearing loss (SSNHL) is a medical emergency; its etiology is unknown in most cases, The treatment, in turn, is empiric and usually consists of various pharmacological agents, mainly steroids. Hyperbaric oxygen (HBO) therapy is used routinely as salvage therapy for refractory SSNHL. While several studies have demonstrated the effectiveness of HBO therapy as salvage treatment for refractory SSNHL, its results have varied among studies, and its efficacy is still unclear.

Aim

We aimed to stratify the effect of HBO therapy as salvage treatment after the failure of steroid therapy for SSNHL.

Method

This is a retrospective case series that involved eight SSNHL patients in the past three years at King Faisal Specialist Hospital & Research Centre (KFSHRC) in Riyadh, Saudi Arabia. Patients' records were reviewed and statistical analysis was performed.

Results

Eight patients were included in this case series; six of them were males, and the mean age of all patients was 46.88 ±20.9 years. One patient had herpes zoster as the cause of SSNHL and seven patients' disease was of unknown etiology. The mean period for the onset of disease was 4.12 ±2.17 days. One patient was managed with intravenous dexamethasone and the other patients were managed with oral steroids, and all patients were then treated by HBO therapy. Of the patients, two showed significant improvement but the others did not. The cause of SSNHL, age of patients, HBO start and cessation, comorbidities, and disease onset did not affect the improvement in disease in patients (p: ˃0.05).

Conclusion

Moderate-to-severe cases of SSNHL can be improved by HBO treatment along with oral steroids, while this therapy was ineffective in severe and profound cases. No factors could be found to predict improvement in patients.

## Introduction

Sudden sensorineural hearing loss (SSNHL) is defined as sensorineural hearing loss to an extent of at least 30 dB, involving not less than three successive frequencies that develop within 72 hours [1]. SSNHL symptoms usually occur suddenly and unilaterally within 24-72 hours [2]. Symptoms include dizziness and tinnitus that occur in 29-56% and 41-90% of patients, respectively [3-6]. SSNHL affects individuals of all ages, but it predominantly occurs in people between the fifth and sixth decade of their lives [7]. It affects almost 5-20 per 10,000 individuals in the US [3,8]. Several pathologic processes have been proposed to be the possible causes of SSNHL, such as autoimmune reaction, viral infection, and circulatory disorders [9]. In the majority of SNHL patients, no specific factor could be identified as the cause of hearing loss; so it was identified as idiopathic [3,4,10,11].

The rate of spontaneous recovery among SSNHL patients ranges from 25-89% [12]. The recovery hearing threshold of patients with SSNHL may be either complete (return to the previous baseline), partial (more than 30 dB improvement), or none at all [7]. Several factors have been found to affect hearing recovery, such as the nature of the onset of hearing loss, the time of the onset of hearing loss, presence of vertigo, age, and frequencies affected [3]. There are different treatment regimens for SSNHL, and the most common one involves steroids. Other regimens may include vasodilators, carbogen therapy, antioxidants, and hyperbaric oxygen (HBO) therapy [2,13]. Steroids can be administrated either by intratympanic injection or systematically via intravenous or the oral route [2]. The recovery outcome rates have been found to be unsatisfactory as the recovery may occur in only 61% of patients [14]. Studies on most of these agents are rare [15]; steroid therapy has shown success in some studies [14,16]. However, other studies did not confirm this success [13,16]. Best results have been achieved when HBO therapy is started within two weeks of the onset in combination with steroids [17]; also, a combination of HBO with systemic steroids was found to be successful in patients with hearing loss of more than 90 dB [18]. Additionally, it was found that an improvement of threshold resulted from HBO therapy especially in those patients with low frequencies with otherwise therapy-refractory SSNHL [19]. The results of HBO therapy for SSNHL treatment vary among different studies. The purpose of this case series is to assess the effect of HBO therapy as salvage therapy after the failure of steroid therapy for SSNHL in patients at King Faisal Specialist Hospital & Research Centre (KFSHRC).

## Materials and methods

Subjects and study design

This was a retrospective case series study that included eight patients who were treated at KFSHRC. These patients had SSNHL and underwent HBO therapy sessions as salvage treatment during the period 2015-2018; we planned to provide 20 to 30 sessions of therapy to these patients. The medical records of the patients were reviewed, and improvement in patients post-HBO therapy was assessed using pure tone audiometry (PTA). Approval from the institutional review board (IRB) was obtained before undertaking the study.

Statistical analysis

Data were analyzed using SPSS Statistics software version 16 (IBM, Armonk, NY). It entailed simple descriptive analysis in the form of numbers and percentages for qualitative variables and median and range for quantitative variables. The Mann-Whitney U test was used to compare quantitative variables between improved and non-improved patients, and Fisher-exact was used as a test of significance to compare qualitative variables. A p-value of <0.05 was considered to be statistically significant.

## Results

The age range of patients was 23-76 years with a mean ±SD of 46.88 ±20.9 years. There were six (75%) men and two (25%) women. There were four patients with comorbidities; one had asthma (12.5%), one had diabetes mellitus (DM), one had hypertension (HTN) and an old cerebrovascular accident (CVA), and one patient had hypothyroidism and dyslipidemia. The cause of SSNHL was unknown in seven patients (87.5%), and only one patient had SSNHL caused by herpes zoster in the form of meningitis. The range of onset duration was one to seven days with a mean ±SD of 4.12 ±2.17 days. The demographics and clinical characteristics of the patients are shown in Table 1.

**Table 1:** Demographics and clinical characteristics of patients SD: standard deviation; DM: diabetes mellitus; HTN: hypertension; CVA: cerebrovascular accident

Characteristics	N (%)
Age (years)	
Mean ±SD	46.88 ±20.9
Median (range)	42 (23-76)
Sex	
Female	2 (25%)
Male	6 (75%)
Comorbidity	
None	4 (50%)
Asthma	1 (12.5%)
DM	1 (12.5%)
HTN, old CVA	1 (12.5%)
Hypothyroidism, dyslipidemia	1 (12.5%)
Cause	
Unknown	7 (87.5%)
Herpes zoster	1 (12.5%)
Onset (days)	
Mean ±SD	4.12 ±2.17
Median (range)	4 (1-7)

Different symptoms were found among patients; three (37.5%) patients suffered from tinnitus, two patients had vertigo, one suffered from otalgia, and only two patients did not experience any symptoms. Five patients were administered with oral steroids. Of them, four started HBO therapy within two weeks; two discontinued HBO therapy after 20 days, one after 16 days, and one after one day. The last patient who was administered with oral steroids started HBO therapy within one week and discontinued it after 15 days. Three patients received different regimens. One was administered with intravenous dexamethasone, and started HBO within two weeks and discontinued it after 20 days. One was administered with an oral steroid and antiviral drugs for herpes zoster, and started HBO therapy within two weeks and discontinued it after 37 days. The last patient received an oral steroid and intratympanic steroid injection, and started HBO after one week and continued for 30 days. Table 2 summarizes the patients' treatment regimen.

**Table 2:** Patients’ regimen of steroids and HBO therapy HBO: hyperbaric oxygen

Steroids	HBO onset	Patients, n (%)	HBO cessation (days)
Oral	Within 2 weeks	4 (50%)	16, 20, 20, 1
Oral	After 1 week	1 (12.5%)	15
Oral steroid and intratympanic steroid injection	After 1 week	1 (12.5%)	30
Intravenous dexamethasone	Within 2 weeks	1 (12.5%)	20
Oral and antiviral	Within 2 weeks	1 (12.5%)	37

PTA was performed before and after the treatment (Table 3). Four (50%) patients had moderate-to-severe SNHL; after treatment, one patient’s case became mild-to-moderate, and only patients (25%) showed improvement. Three (37.5%) cases were profound and they did not show improvement after treatment. One patient (12.5%) suffered from severe SNHL and showed no improvement after treatment.

**Table 3:** Clinical investigation and patient improvements PTA: pure tone audiometry; SNHL: sensorineural hearing loss

Before PTA	After PTA	Patients, n (%)	Outcome
Moderate-to-severe SNHL	Mild-to-moderate SNHL	4 (50%)	2 improved
			2 showed no improvement
Profound	Profond	3 (37.5%)	3 showed no improvement
Severe	Severe	1 (12.5%)	1 showed no improvement

The influence of selected factors on patient recovery was investigated; however, none of them were found to be associated with improvements in patients. These factors included the cause of the disease, time of onset and cessation of HBO, presence of comorbidities, age, the onset of the disease, associated symptoms, or degree of hearing loss (p: ˃0.05) (Table 4).

**Table 4:** Factors influencing patient improvement *n (%): for qualitative variables; median (range): for quantitative variables. **Fisher’s exact test HBO: hyperbaric oxygen

Factors	Improved, n (%)/median (range)*	Not improved, n (%)/median (range)*	P-value**
Cause			1
Unidentified	2 (28.6%)	5 (71.4%)	
Herpes zoster	0 (0%)	1 (100%)	
HBO onset			
After 1 week; within 2 weeks	1 (50%); 1 (16.7%)	1 (50%); 5 (83.3%)	0.46
Comorbidity			1
No	1 (25%)	3 (75%)	
Yes	1 (25%)	3 (75%)	
Age (years)	55.6 (45-66)	35 (23-76)	0.6
Onset (days)	4.5 (2-7)	4 (1-7)	0.8
Cessation (days)	25 (20-30)	18 (1-37)	0.4
Associated symptoms			0.464
Yes	1 (50.0)	1 (16.7)	
No	1 (50.0)	5 (83.3)	
Degree of hearing loss			0.107
Severe or profound	0	5 (83.3)	
Mild to moderate	2 (100.0)	1 (16.7)	

## Discussion

The present case series included eight patients; six of them were males and two were females. The age range of the patients was 23-76 years with a mean age of 46.88 years. Half of the patients did not suffer from any comorbidity; the range of the onset duration was one to seven days with a mean of 4.12 days. It has been reported previously that the incidence of SSNHL is found equally in males and females [3,4,10,11]. However, SSNHL in our case series was more dominant in males than in females. Different pathologic processes have been suggested to be the possible causes of SSNHL, including viral infections; however, in most cases, the cause remains unknown [9,15]. In the current case series, there were seven patients with unknown cause of SSNHL, while only one patient had a known cause, with herpes zoster being the cause of SSNHL occurrence. Seven patients in this case series were administrated oral steroids and only one patient was administrated intravenous dexamethasone. The patients were then started on HBO therapy; however, two patients who were administrated steroid therapy were also given other agents. An antiviral agent was administrated to one patient who had herpes zoster, and the other patient was managed with an intratympanic steroid injection in addition to oral steroids. It has been reported that the best results can be obtained by starting HBO therapy within two weeks in combination with steroids [17]. Six of our patients started the HBO therapy within two weeks, and only two patients started HBO after one week.

The duration of HBO administration ranged from 1-37 days. It was found that the administration of HBO therapy besides the primary conventional therapy improved outcomes of patients when started early [20,21]. The improvements in our patients were investigated; patients who had profound SSNHL (three patients) before treatment regimen did not show any improvements and their status remained profound. The same was found for the case that suffered severe SSNHL; the treatment did not lead to any improvement. There were four patients who suffered moderate-to-severe SSNHL before treatment, and after treatment, two patients improved and two did not. In Bal et al.'s case study, it was demonstrated that the combination of both intratympanic steroid and HBO therapy with oral steroids may enhance the efficiency of treatment and the patient can be rescued from cochlear implementation surgery [22]. Another case report by Imsuwansri et al. found that a patient with idiopathic SSNHL did not respond to intratympanic and systemic steroid treatment, but he recovered after HBO therapy [23]. One study [15] showed that using HBO as salvage therapy for SSNHL resulted in improvements in those with severe hearing loss, whereas patients with mild or moderate hearing loss can recover at low frequencies; however, we found the opposite in the current work. Another study [24] recommended HBO therapy for the treatment of SSNHL. In a prospective controlled study, it was found that significant improvements were obtained by the application of HBO therapy in patients whose corticosteroid treatment failed [23]. Another study supported the previous findings by suggesting HBO therapy for all patients who did not respond to conventional therapy, and the improvement rate was 46% among the study group [24]. It was reported that recovery may reach up to 61% among patients [14]; however, among our patients, only 25% have recovered (two patients). It is reported that there are several factors that impact hearing recovery, including hearing-loss severity, age at the time of the onset of hearing loss, and time between the onset of hearing loss and treatment [3]. However, in this case series, the improvements in patients were not affected by any of the following factors: the cause, start and cessation of HBO, presence of comorbidity, age, disease onset, associated symptoms, or degree of hearing loss. Another previous study has shown that hearing improvements were not associated with age [15], which is consistent with our findings.

## Conclusions

Based on our findings, moderate-to-severe SSNHL cases can be improved with treatment by a combination of steroid and HBO therapy, while profound and severe cases showed no improvement. We could not identify any factor that influenced the improvements in our patients as there was no significant difference between patients who showed improvement and those who did not with respect to factors suggested by previous studies.
